# Gamma-Band Modulation in Parietal Area as the Electroencephalographic Signature for Performance in Auditory–Verbal Working Memory: An Exploratory Pilot Study in Hearing and Unilateral Cochlear Implant Children

**DOI:** 10.3390/brainsci12101291

**Published:** 2022-09-25

**Authors:** Bianca Maria Serena Inguscio, Giulia Cartocci, Nicolina Sciaraffa, Maria Nicastri, Ilaria Giallini, Antonio Greco, Fabio Babiloni, Patrizia Mancini

**Affiliations:** 1 Department of Sense Organs, Sapienza University of Rome, Viale dell’Università 31, 00161 Rome, Italy; 2 BrainSigns Srl, Lungotevere Michelangelo, 9, 00192 Rome, Italy; 3 Department of Human Neuroscience, Sapienza University of Rome, Viale dell’Università 30, 00161 Rome, Italy; 4 Department of Molecular Medicine, Sapienza University of Rome, Viale Regina Elena 291, 00161 Rome, Italy; 5 Department of Computer Science, Hangzhou Dianzi University, Xiasha Higher Education Zone, Hangzhou 310018, China

**Keywords:** working memory, deafness, cochlear implants, children, EEG, gamma, theta, n-back, verbal audio, verbal video

## Abstract

This pilot study investigates the neurophysiological patterns of visual and auditory verbal working memory (VWM) in unilateral cochlear implant users (UCIs). We compared the task-related electroencephalogram (EEG) power spectral density of 7- to 13-year-old UCIs (*n* = 7) with a hearing control group (HC, *n* = 10) during the execution of a three-level n-back task with auditory and visual verbal (letters) stimuli. Performances improved as memory load decreased regardless of sensory modality (SM) and group factors. Theta EEG activation over the frontal area was proportionally influenced by task level; the left hemisphere (LH) showed greater activation in the gamma band, suggesting lateralization of VWM function regardless of SM. However, HCs showed stronger activation patterns in the LH than UCIs regardless of SM and in the parietal area (PA) during the most challenging audio condition. Linear regressions for gamma activation in the PA suggest the presence of a pattern-supporting auditory VWM only in HCs. Our findings seem to recognize gamma activation in the PA as the signature of effective auditory VWM. These results, although preliminary, highlight this EEG pattern as a possible cause of the variability found in VWM outcomes in deaf children, opening up new possibilities for interdisciplinary research and rehabilitation intervention.

## 1. Introduction

It is well known that the relationship between hearing and language development is essential in the early years of a child’s life (i.e., [1,2]). The perception, development and use of human language are firmly based on an acoustically transmitted signal [3,4]. Therefore, even a slight hearing loss negatively affects language development in children and delays the acquisition of language, social, academic, and sensory skills [5]. Furthermore, as language and speech development are prerequisites for cognitive development, a hearing defect may influence and impair the cognitive abilities of deaf and hard-of-hearing (DHH) children [6].

The benefits of cochlear implantation for restoring hearing and supporting the development of communication skills in prelingually deaf children are firmly established [7,8,9], especially when using basic clinical measures of speech recognition outcomes. However, there is a tremendous degree of variability and individual differences in the effectiveness of cochlear implants (CIs) [10]. In fact, despite children who receive a CI early in life generally performing better on a wide range of speech and language outcome measures than children who receive a CI at older ages [11,12], significant variability in speech and languages outcomes are routinely observed in this clinical group, and a subgroup of children with CIs fail to attain optimal speech–language outcomes [12,13]. Researching the precise causes of this variability, the extensive studies of the Pisoni and Kronenberger group showed that CI users are at risk of delays in developing Executive Functions (EFs) [8,14,15,16]. EFs are referred to as higher-order cognitive processes that enable, for instance, to flexibly set up and monitor goal-directed behaviors like attention and regulation, especially in complex circumstances [17,18]. Children with CIs showed greater rates of delay than hearing peers in multiple subdomains of EFs. However, it is in verbal working memory (VWM)—commonly defined [19] as the temporary maintenance of verbal information (i.e., some aspects of language)—that the most significant and most consistent delay, compared to hearing peers, has been found [8,20,21,22,23,24], regardless of the modality of the verbal stimuli presentation [25]. As a result, VWM has been identified as a fundamental domain of neurocognitive risk and a potential target for intervention to enhance speech–language outcomes in CI users [24]. However, a comprehensive understanding of how these factors interact and the neural mechanisms underlying these interactions are unknown. In fact, most studies have addressed working memory (WM) deficits in children with cochlear implants by assessing outcomes with psychometric scales and speech tests [26,27], especially in Verbal WM, and investigation of the neural correlates of this crucial executive function in children is rare, particularly in clinical children’s samples [28].

Early sensory experiences shape the neural circuitry of the auditory system, and there is a pronounced reduction in synaptic plasticity in the auditory cortex during early deafness [29]. Electrophysiological studies have shown that early access to sound with CI can mitigate some structural and functional effects of congenital deafness on the cortical auditory system [30], which are likely to change the cortical network involved in hearing [31,32]. However, CIs provide poor sound encoding in terms of frequency selectivity and temporal encoding if compared to hearing subjects [33]. These interventions are thus are unlikely to completely restore the normal auditory connectome, even in children who receive implants in both ears at young ages. So, as Kral and Sharma [34] pointed out, a more comprehensive understanding of the neural correlates of individual variability will be critical to developing better rehabilitation options that are aimed at and customized for individual patients. Moreover, neuroimaging studies have broadly observed cross-modal plasticity in DHH subjects with CI [35,36]. For example, Song and colleagues, in a positron emission tomography (PET) study [37], found greater visual activation for audiovisual speech in CI users, suggesting a plastic effect on multimodal perception, inferring an incomplete reversal of cross-modal plasticity after hearing restoration, or that auditory reorganization is maintained by a continued reliance on visual input [38]. In a functional magnetic resonance imaging (fMRI) study with early deaf adults, auditory regions showed greater cross-modal activation during a visuospatial WM task in clinical subjects than in controls. Furthermore, cross-modal activation in the auditory areas correlated with WM performance in deaf but not in hearing participants [39]. Considering these findings and the previous literature on left-hemispheric lateralization of WM in hearing subjects [40,41], the question arises as to the lateralization of VWM function and whether or not it is dependent on the sensory modality of stimulation in DHH children.

Few recent studies have investigated cortical activations through electroencephalography (EEG), a powerful, accessible and versatile neuroimaging tool for investigating human brain physiology, cognition and behavior [42,43] in CIs children during cognitive tasks. For example, researchers used theta and alpha connectivity to differentiate performance across different implant processors [44] or hemispheric gamma activation to suggest the occurrence of a sensitive period for CI surgery for best emotion recognition skills development [45]. Moreover, during a listening task in the noise, a higher workload index (theta/alpha) in over-demanding situations is recorded [46], as well as displaying lower parietal alpha power levels in the most challenging listening condition [47]. Furthermore, alpha oscillations appear particularly sensitive to hearing loss during WM paradigms [48]. Finally, Cartocci and colleagues [49] showed a correlation between the period of deafness and the cortical activity asymmetry toward the hearing ear side in the frontal, parietal and occipital areas. These findings are in accord with several studies reporting that strain on cognitive resources for auditory perception leaves less availability for cognitive processing [50,51,52] and converge toward the hypothesis that the cause of the wide variability found in deaf children with cochlear implants in VWM may lie in scalp-recorded neural oscillations. Indeed, there has been considerable interest in determining the locus of outcome variability in children with CI in recent years. Researchers have identified that cognitive factors, such as WM, are critical in the healthy development of children with CI, and despite the effect of CI-treated profound hearing loss on auditory and visual–verbal WM performance, its neural correlates remain unclear [48,53]. In particular, to our knowledge, the EEG power spectrum related to the audio-visual WM task has not been explored in children CI users.

### The Aim

In light of the scientific evidence available in the literature to date, the present study aims to investigate the impact of unilateral cochlear implant use in deaf and hard-of-hearing (DHH) children on the neurophysiological patterns underlying VWM processing of auditory and visual stimuli during an n-back task. The pioneering goal of the present study, which is comparing the EEG signals of DHH children unilateral CI users with a control group of hearing children, is to reveal the neurophysiological sensorial patterns of VWM. This is in order to explain the extreme behavioral variability found in this clinical group concerning this essential cognitive function. Findings could support an improved clinical and rehabilitative approach for these patients.

## 2. Materials and Methods

Methods for recording and analysis in the current study follow those described in previous publications from our group [28,45]. An abbreviated version of these methods is provided below.

### 2.1. Participants and Ethics Statement

The sample size was determined by a power analysis before data collection using G*Power (Universität Düsseldorf, Düsseldorf, Germany) [54]. Given the preliminary nature of the present study, seven right-unilateral cochlear-implanted children (UCI, mean age 11.22 years ± 0.63 SD) and ten age-matched hearing children (HC) were recruited. Still, this went beyond the minimum 10% of the total sample requested for pilot studies [55]. Demographic and clinical data for the UCI group are summarized in Table 1. The eligibility criteria for the clinical group included congenital severe/profound deafness (Pure Tone Average in the better ear ≥ 80 dB HL for 500–4000 Hz), good speech perception abilities, defined as bisyllabic word recognition and sentence comprehension >90% in a silent room at the moment of the EEG test; none of UCI wore any hearing aid in the contralateral ear to the one with the cochlear implant. The age of the sample was determined according to previous studies [56,57]. Raven’s standard progressive matrices (RPM) [58], a test of non-verbal spatial reasoning, was used for the screening for the participant selection. Exclusion criteria for enrolment in the study were diagnosis of neuropsychiatric disorders and/or sensory deficits; children with scores below the standard average for their age (taken from test norm) on RPM; left-handed children due to past evidence of handedness influence on cerebral laterality [59]. Before the experiment, participants and their parents were fully informed about the study. The investigation was conducted according to the principles outlined in the Helsinki Declaration of 1975, revised in 2000 and approved by the Institutional Ethics Committee of Policlinico Umberto I—Rome, Italy (no. 259/2020). Informed written consent was obtained from all parents before the protocol started. Participation in the study was voluntary; children received a present after their involvement.

### 2.2. Overview of Experimental Design and Procedure

During the EEG recording, participants performed two verbal n-back tasks [60] with different memory loads from 0-back to 2-back: an auditory n-back task (AUD-task) in which stimuli were presented aurally, and a visual n-back task (VIS-task) in which stimuli were presented visually. The order of the task administration and the order of the n-back blocks presentation were randomized across participants.

*Stimuli*: verbal material consisted of auditory and visual stimuli, referring to seven consonants (c, g, k, p, q, t, v) already used in previous experimental protocols [61,62,63,64]. Vowels were excluded to reduce the likeliness of participants developing chunking strategies [65]. To ensure correct perception by UCI and HC groups, we performed a stimuli exposure pretest. Visual stimuli (Consolas font—130) with a duration of 500 ms and an interstimulus interval of ISI 3000 ms [56] were presented one at a time on a grey background in the center of a monitor screen placed at eye level, 50 cm from the participant. Auditory stimuli (duration 500 ms; ISI 2500) [61] consisted of a recorded female voice, set at a 65 dB SPL intensity to ensure comfortable audibility to both HC and UCI [45], transmitted by two audio speakers placed at 45 degrees left/right, at face level 1 m in front of the participant.

*Task execution*: Immediately after the stimuli presentation, participants in the ISI had to respond by pressing a previously reported key (D/K) on the keyboard to indicate whether the letter was a target (K) or a nontarget (D): thus, there was a behavioral response in either case. In the 0-back condition, the letter X was the target. In the 1-back condition, a letter was a target when it was the same as the one presented immediately before. In the 2-back condition, a letter was a target when it was the same as the two letters before. Participants received detailed instructions on how to perform the task correctly and a training session was undertaken before the practical measurement session to familiarize them with the experimental procedure.

**Figure 1:**
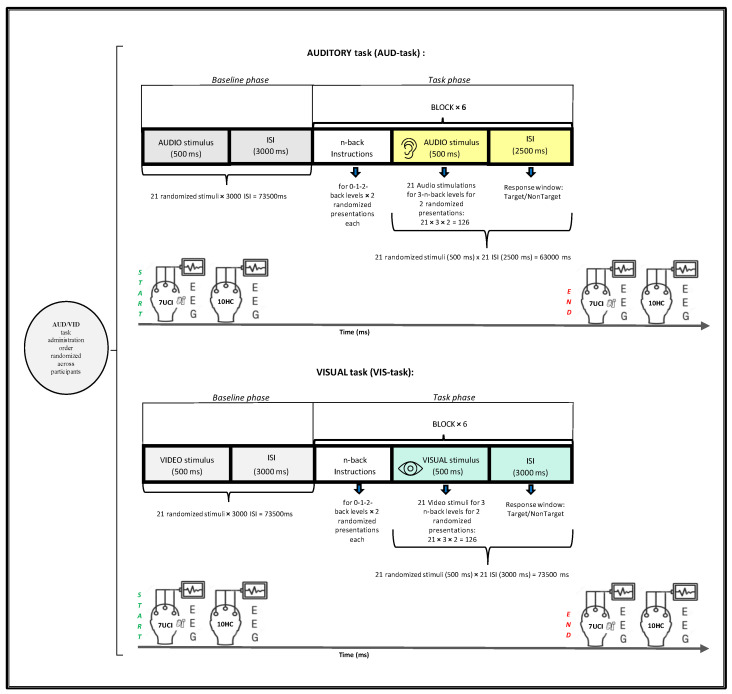
Experimental design with the trial timeline. Schematic illustration for each n-back task (auditory-AUD and visual-VIS modalities) performed by hearing children (HC) and unilateral cochlear implant (UCI) groups during electroencephalography (EEG) recording. Each modality task started with the baseline phase, followed by the task phase.

*Task structure*: load levels (0, 1, 2-back) were presented in six blocks (2 for each level) for each task (auditory and visual). The blocks consisted of 21 randomized stimuli (30% target) [56]. A baseline phase, during which participants were asked to remain relaxed with no task except to look at the screen while auditory or visual stimuli were presented, anticipated the task phase. During the baseline phase, the 7 stimuli were repeated randomly 3 times (500 ms with 3000 ms ISI), creating a 21-item block analogous to the experimental blocks. The task phase then consisted of 2 randomized presentations of the three blocks. Thus, each session consisted of 3-n back levels per 2 presentations for 6 blocks in randomized order for audio and visual tasks. Half of the participants started with the visual stimuli task, and the other half with the auditory task (see Figure 1 for a visual task structure synthesis). A Lenovo PC (monitor resolution 1024 × 768) displayed and controlled stimuli presentation and collected participants’ responses in terms of reaction times (RTs) and correct responses (CRs) through the software package E-Prime (Psychology Software Tools, Pittsburgh, PA, USA, Version 3.0).

*Procedure*: the participant was seated on a chair in an audiometric test room while the experimental design was fully explained. Participants were instructed to assume a comfortable position and avoid unnecessary movement to reduce muscular artefacts in the EEG signal. After each task phase, the participant indicated the perceived task difficulty (easy–medium–hard) on a stylized image; at the end of the entire experimental session, they were asked to rate which of the two tasks (visual or auditory) was the most difficult.

### 2.3. Behavioral Measures

Performances were evaluated in terms of accuracy (ACC), calculated as the percentage of correct responses for each task condition (each n-back level for auditory and visual modality tasks). To integrate the correct answers and the reaction times (RTs, measured from the time of stimulus offset) for each response, inverse efficiency score IES = RT/1-PE was calculated, where RT is the subject’s average RTs for correct answers (target/nontarget). PE is the subject’s proportion of errors for each condition. IES can be interpreted as the RT corrected for the number of errors committed [66].

### 2.4. EEG Recording and Signal Processing

To record 20 EEG channels (Fpz, Fz, F3, F4, F7, F8, Cz, C3, C4, T7, T8, Pz, P3, P4, P7, P8, Cp5, Cp6, O1 and O2) referred to the participants’ earlobes, a digital ambulatory monitoring system (BePlus System-EBNeuro, S.p.A., Firenze, Italy) with a sampling frequency of 256 Hz was used. The impedance was kept below 10 kΩ, and a 50 Hz notch filter was then applied to remove power interference. EEG signals were initially band-pass filtered with a 5th order Butterworth band-pas filter (1–45 Hz) to reject continuous components and high-frequency interferences like such as muscular artefacts. The Fpz channel was used to eliminate eye-blink contributions by the REBLINCA algorithm [67] without losing data. Specific procedures of the EEGLAB toolbox (Schwartz Foundation, Halesite, NY, USA) [68] were used to depurate from other artefacts. The EEG dataset was segmented into epochs starting 500 ms before stimulus onset and ending 2500 ms after the offset. This temporal windowing was adopted to respect stationary EEG and allow for a high number of observations, compared to the number of variables considered in the analysis [69]. To identify artefacts, three criteria were employed according to published procedures [45,49,70]: (i) threshold criterion (±80 µV); (ii) trend estimation criterion (slope higher than 40 µV/s or less than 0.3 µV/s); (iii) sample-to-sample criterion (when, in terms of absolute amplitude, the signal sample-to sample >30 µV/s). Finally, all epochs marked as “artefacts” were removed from the EEG dataset, such that all analyses were based on clean EEG signals. To accurately define EEG bands of interest, individual alpha frequency (IAF), given in Hertz, was computed for each participant on a 60 s long-closed eyes segment, recorded before the baseline phase [71]. Each band was then defined as IAF± x, where x was an integer in the frequency domain; thus, the EEG signal was filtered in the following frequency bands in Hertz (Hz): theta [IAF − 6 ÷ IAF − 2 Hz], alpha (IAF − 2 ÷ IAF + 2 Hz), beta (IAF + 2 ÷ IAF + 16 Hz), and gamma (IAF + 16 ÷ IAF + 30 Hz) [71]. Then, the power spectral density (PSD) [72] was calculated for each epoch and channel, with a Hanning window of 1 s and an overlap of 500 ms. Topographical distribution of band modulation analysis was based established on averages of the data for the following areas of interest (AOIs): frontal, parietal, occipital and hemispheres electrode locations. The channels considered were F3, F4, Fz (frontal); Pz, P3, P4, P7, P8 (parietal); O1, O2 (occipital); F3, C3, T7, P3, O1 (left hemisphere); F4, C4, T4, P4, O2 (right hemisphere). In addition, the Workload Index (WI) was calculated in accordance with the formula given above.To limit bias on scores due to subjective stimuli perception on VWM n-back task recording, PSD data were normalized with respect to the baseline [73].

### 2.5. Statistical Analysis

Both neuro and behavioral data were objects of statistical analysis in this study. The Shapiro–Wilk normality test [74] was applied to the dataset under investigation. Then, depending on the results, parametric or non-parametric analysis of variance (ANOVA) was performed [75]. Both behavioral (ACC; IES) and neurophysiological (AOIs; WI) values were entered in a 2 × 3 factorial ANOVA with two factors: factor modality (with 2 levels: audio and video) and factor load (with 3 levels: 0-1-2). Duncan’s post hoc test [76] was used to investigate statistically significant results of ANOVA tests; partial eta squared ($\eta_{p}^{2}$) effect sizes were reported [77,78]. The ANOVA test has sufficient statistical power to deal with the analysis of relatively small numbers of participants, as in this study [79], provided that the number of factors is lower than 4, as in this case. A correlation analysis was performed to assess possible relationships between variables, while simple regression analysis was used to investigate potential functional relationships between variables (e.g., mean of tot audio; mean of total 2 back). A cut-off of α = 0.05 was set as the cut-off of significance [80].

## 3. Results

### 3.1. Behavioral Results

The overall ACC percentages were greater during all auditory n-back levels and increased with decreasing memory load for both HC and UCI. Furthermore, IES scores for both groups were higher during all levels of auditory n-back and decreased with decreasing memory load compared to visual n-back (Table 2).

**Figure 2:**
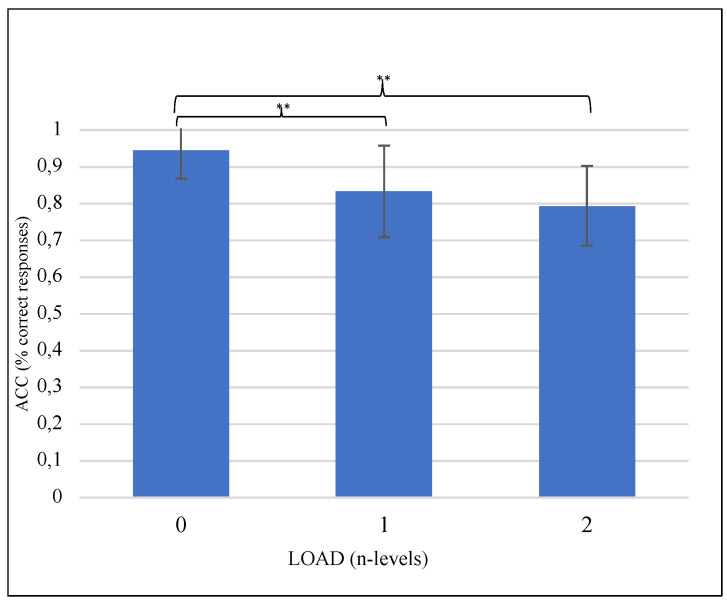
The graph shows the significantly different ANOVA behavioral results of performances in terms of accuracy (ACC) expressed as % of correct responses according to LOAD condition (the 3 levels of the n-back verbal working memory task). Significant differences between load conditions emerging from the post hoc test are indicated (** *p* ≤ 0.01).

ANOVA results in terms of ACC showed a statistically significant difference between memory load (F(2,30) = 23.992, *p* < 0.001, $\eta_{p}^{2}$ = 0.615). The highest significant percentages of correct responses were measured for n0 compared to n1 (*p* < 0.001) and n2 (*p* < 0.001) load conditions (Figure 2).

**Figure 3:**
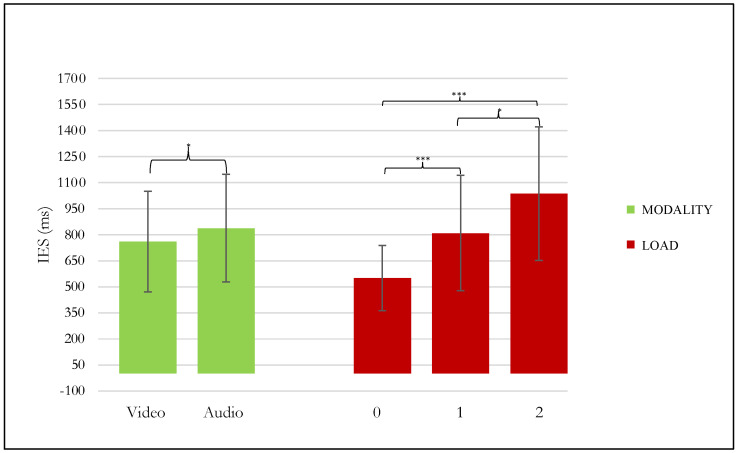
The graph shows the significantly different ANOVA behavioral results of inverse efficiency score (IES) in milliseconds (ms) according to LOAD (the 3 levels of the n-back verbal working memory task) and MODALITY (video and audio) conditions. Significant differences between verbal working memory (VWM) load and modality conditions emerging from the post hoc test are indicated (* *p* ≤ 0.05; *** *p* ≤ 0.001).

ANOVA results showed a statistically significant effect of both LOAD (F (2,30) = 30.351, *p* = 0 < 001, $\eta_{p}^{2}$ = 0.669) and MODALITY (F (1,15) = 5.8843, *p* = 0.028, $\eta_{p}^{2}$ = 0.281) and a significant interaction between these two factors (F (2,30) = 7.3515, *p* = 0.002, $\eta_{p}^{2}$ = 0.328) on IES. Post hoc analyses revealed significant increases in IES, respectively, both as the difficulty increased: from level 0-back to level 1 and 2-back (*p* < 0.001 resp.) and between level 1 and 2-back (*p* = 0.024) and for the auditory compared to visual modality (*p* = 0.028) (Figure 3). Furthermore, the 2-audio condition produces significantly higher IES values than all task conditions (*p* < 0.001).

### 3.2. Neurophysiological Results

**Figure 4:**
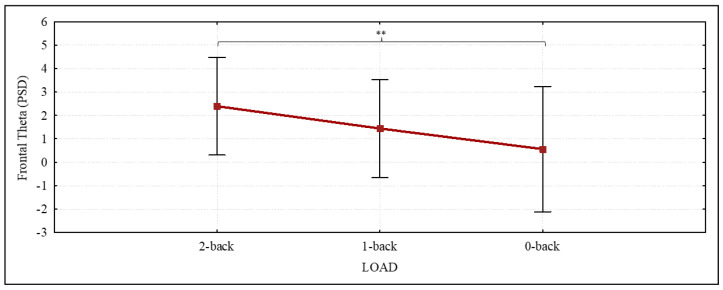
Theta band results. The graph shows the significantly different ANOVA power spectral density (PSD) theta results in the frontal area in relation to verbal working memory (VWM) n-back task levels (LOAD). Significant differences emerging from the post hoc test are indicated (** *p* ≤ 0.01).

When the EEG theta band was considered, there was a significant effect of the LOAD condition on power values (F (2,30) = 4.852, *p* = 0.014, $\eta_{p}^{2}$ = 0.244) in the frontal area. In this case, post hoc Duncan’s test detected a significant difference between 2-back and 0-back levels (*p* = 0.002) (Figure 4).

**Figure 5:**
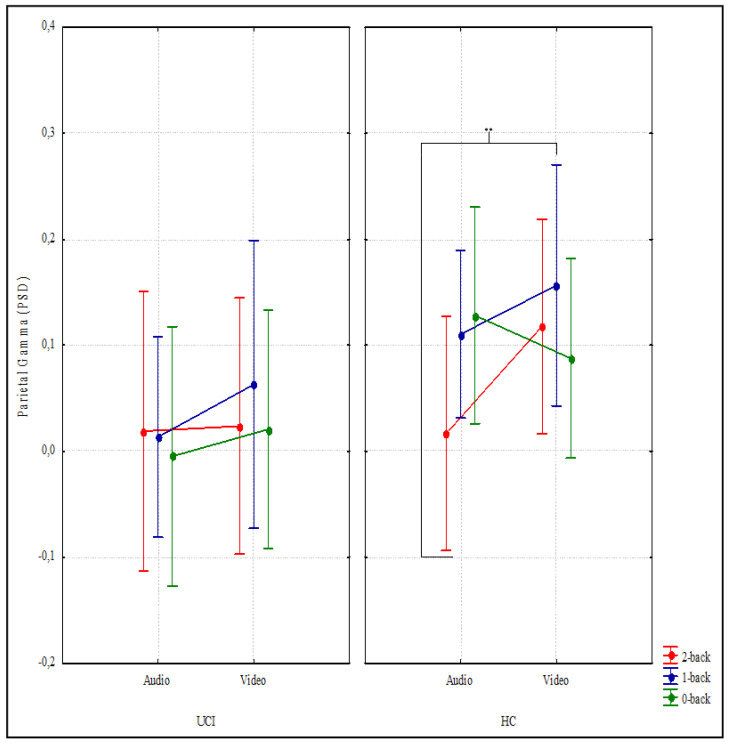
Gamma band results. The graph shows the significantly different ANOVA power spectral density (PSD) gamma results in the parietal area in relation to LOAD (n-back task levels) × MODALITY (Audio and Video) × GROUP (HC = hearing children; UCI = unilateral cochlear implanted children). Significant differences between conditions emerging from the post hoc test are indicated (** *p* ≤ 0.01).

Concerning gamma activity, the analyses in the parietal area showed considerable interaction between LOAD × MODALITY × GROUP factors (F (2,30) = 3.499, *p* = 0.043 $\eta_{p}^{2}$ = 0.189). Post hoc analyses showed a significant difference solely within HC between condition 2 audio and all other conditions (*p* ≤ 0.001) except for the 0-video condition (Figure 5).

**Figure 6:**
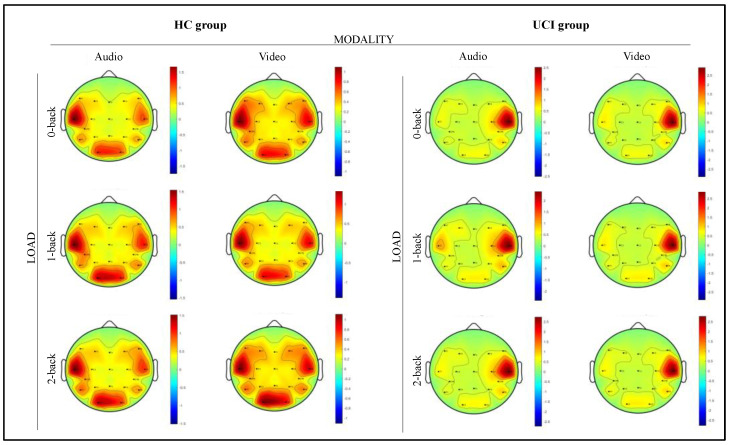
Scalp distribution of the Electroencephalographic (EEG) gamma spectral power during the audio and video n-back tasks. Taped to the left is the gamma activation of the hearing control (HC) group, and to the right, those of the unilateral cochlear implant (UCI) group. For each group from left to right, the scalp maps correspond to the MODALITY (audio and video) and LOAD (0, 1, 2 level) n-back verbal working memory (VWM) task conditions. The black dots correspond to the electrode positions.

Gamma activity also, regardless of conditions, was greater at the limit of significance in the left hemisphere than in the right (F (1,15) = 4.165 *p* = 0.059, $\eta_{p}^{2}$ = 0.270). At the same time, this activation pattern in other frequency bands was not observed. Delving into the different hemispheric activation between groups based on bands, solely for gamma, a marginally significant difference is observed (F (1,15) = 4.4381, *p* = 0.052, $\eta_{p}^{2}$ = 0.228), revealing a significantly greater activation in the left hemisphere for HC compared to UCI (see Figure 6 for the global activations in gamma frequency).

**Figure 7:**
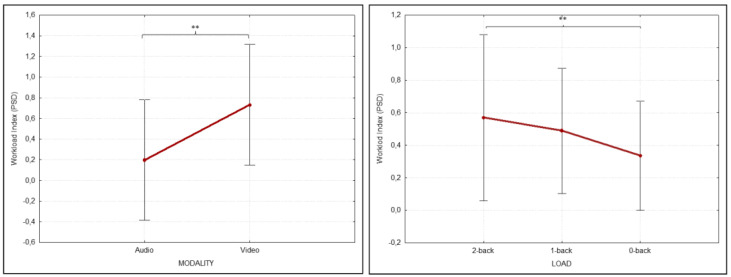
Workload Index (WI) results. Significantly different ANOVA power spectral density (PSD) WI results in relation to both MODALITY (**left**) and LOAD (**right**) conditions. Significant differences between conditions emerging from the post hoc test are indicated (** *p* ≤ 0.01).

In terms of WI, the analysis showed the effect of both LOAD (F (1,15) = 8.679 *p* = 0.010, $\eta_{p}^{2}$ = 0.206) and MODALITY (F (1,15) = 8.679 *p* = 0.010, $\eta_{p}^{2}$ = 0.366) factors. Post hoc analysis highlighted increased WI values comparing 2-back and 0-back levels (*p* = 0.002) and during video versus audio presentation (*p* = 0.008) (Figure 7).

**Figure 8:**
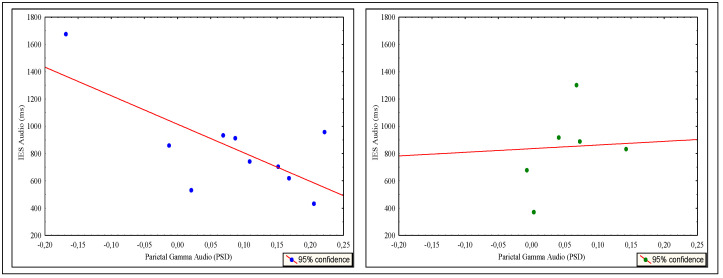
Scatterplot of auditory inverse efficiency score (IES) as predicted by parietal gamma activation across the hearing control (HC) group (*n* = 10) (left, blue dots) and not predicted across the unilateral cochlear implanted (UCI) group (*n* = 7) (right, green dots). Simple linear regression explained 50.6 % of the variance in IES performance based on parietal gamma activity during the auditory n-back task for the HC group.

Correlation analysis showed significant relationships between parietal activation for audio conditions in gamma band and audio IES in HC (r = −0.71), while in UCI, a correlation is observed only between parietal gamma activation with current age for audio condition and not with behavioral data (r = 0.86). Furthermore, during the audio task, always considering the limitations of the analysis due to the numerosity and variability of the group, the simple linear regression analysis demonstrated a significant linear dependence between gamma oscillations in the parietal area and IES only for HC participants (R^2^ = 0.506, R^2^ adjusted = 0.445) and during the audio task (Figure 8).

**Figure 9:**
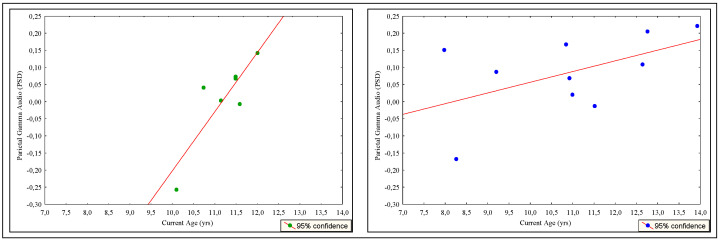
Scatterplot of parietal gamma activation as predicted by current age across the unilateral cochlear implant (UCI) group (*n* = 7) (left, green dots) and not predicted across the hearing control (HC) group (right, blue dots). Simple linear regression explained 74.10% of the variance in parietal gamma activation based on the current age auditory n-back task for the UCI group.

Inversely, UCI participants showed a linear dependence between current age and EEG activation in the gamma band during the audio task (R^2^ = 0.741, R^2^ adjusted = 0.689) (Figure 9).

## 4. Discussion

In the current pilot study, we analyzed behavioral and neural correlates of VWM processing during auditory and visual n-back tasks in normal hearing and DHH children unilateral cochlear implant users. To date, these measures have rarely been used in UCI children while being reported for the visual n-back task in hearing adult and children’s groups (i.e., [57,64,81,82]). Primarily, this study is the first one in which the same participants (HC and DHH) conducted a verbal n-back task in two sensory modalities (auditory and visual) while EEG data were recorded. This allowed us to compare the typical load-related EEG (i.e., alpha, theta, gamma) and behavioral measures for two different tasks and between normal hearing and deaf groups. Overall, we expected these measures to show significant differences between groups to explain the extreme VWM behavioral variability found in children receiving cochlear implant.

### 4.1. Behavioral Results

Predictably, performance in terms of accuracy (ACC) worsens as the VWM load increases (Figure 2) in line with the general literature on n-back tasks children’s performances [56,63]. Furthermore, the sensory modality does not influence performances in both groups. In fact, in contrast to the prior research that consistently demonstrated poorer VWM skills in children CI users [24,83], no effect of group membership is observed on both accuracy and IES measures. However, it is essential to note that VWM in the studies mentioned above was individually assessed by batteries of psychometric tests (i.e., subtest of WISC-V). Whereas, in the present study, we used an n-back task, a handy tool for the investigation of the WM process, especially in children [56]. Therefore, since the same assessment instruments were not used, the direct comparison of the behavioral data cannot have absolute reliability. Moreover, both HC and UCI groups have higher IES values in the audio conditions, especially for 2-back, which seems to be the most challenging task condition [84] regardless of the group (Figure 3). Longer IES values for the auditory task are in line with previous studies on HC children and adults [28,85,86,87], and contradict the hypothesis that auditory stimuli enhance performance by having longer-lasting representation [88,89] and more durable stimuli binding [90]. This result seems particularly interesting regarding the clinical group, given that the model of Pisoni and colleagues [23] proposes that poor performance on VWM tasks in CI users could be partly due to fragile, underspecified phonological representations of letters in short-term memory. Globally, behavioral VWM performances were not different between CI users and hearing controls. However, evidence suggests that performance differences between VWM tasks in post-lingually deafened CI users (both adults and children) and HC are mixed [91,92]. For example, our results are consistent with those reported by [48] during a visual WM task in adults. Furthermore, the latter involved only visual stimuli and concerned an adult sample. Therefore, the lack of differences in performance with DHH children and the absence of significant correlation with the auditory age would preliminarily confirm the cochlear implant’s effectiveness in supporting deaf children’s VWM performance during an n- back task, regardless of the modality of stimulation.

### 4.2. Neurophysiological Results

#### 4.2.1. Workload Index

Mental workload (WL) is a fundamental concept in the study of human performance, emerging from the observation that our cognitive system has a limited capacity to perform a cognitive task [93,94]. WL emerges from the interaction between the task at hand and the individual with limited resources [95]. A previous study reported a WL index (WI) modulation in CIs children during the most challenging noise condition in a forced-choice word recognition task [46]. It is, therefore, to be expected that CI users would show a higher WI in the auditory than in the visual n-back task in light of their clinical condition.

To infer the mental state of cognitive load in VWM, we combined the neurophysiological WI [71,96] with a self-report measure of subjective difficulty. During the n-back task, the WI estimate reflected in part the trend of the behavioral IES score. In fact, WI presented the highest values during the 2-back and the lowest values during the 0-back, respectively, the most and least challenging WM conditions, with no differences between the HC and UCI groups (Figure 7). Furthermore, the increased WI in the video condition compared to the audio condition (Figure 7) reflected the total perception of self-reported difficulty by 52.94% of the global participants. Moreover, while self-reported difficulty was equally distributed between auditory and visual modalities in the HC group, 57.142% of the UCIs stated that they perceived more difficulty with the visual task than for the auditory task. Our findings, although preliminary due to the numerosity of the observed samples, give evidence of the goodness of WI as a brain measure of cognitive load also in a clinical context, being usually used in non-clinical contexts e.g., [97,98] managing to reflect the self-reported difficulty-of-perception measure, particularly in CI users.

#### 4.2.2. Theta

Attentional processes activate engage frontal areas involved in the generation of theta oscillations [71,99,100]. The present study results (Figure 4) showed greater pronounced frontal theta power associated with increased n-back task complexity and are reminiscent of several other studies describing theta activity in humans performing WM tasks [99,101]. Our findings could be explained by the increased attention [102,103,104] in correspondence to the experimental situation that was characterized by higher memory load and/or effortful cognitive processes in HC [105] and unilateral deafness children [46]. However, frontal theta activation analysis showed non-altered activity in UCI compared to controls and global a-modal processing of VWM in both groups. Moreover, it is possible to suggest that children CI users have developed adequate abilities and efficient strategies for allocating more attentional resources to support performance in a complex VWM task regardless of sensory modality, thus bridging the sensory gap due to their natural condition.

#### 4.2.3. Gamma

Hemispheric functional specialization or functional asymmetry is a well-established characteristic of functional organization in the human brain. Verbal sound processing predominantly occurs in the left hemisphere, whereas nonverbal sound processing predominates in the right hemisphere [106]. Studies of the performance of brain-damaged children provide less clear evidence regarding functional asymmetry because such results are frequently confounded with the effects of neural plasticity [107]. Moreover, previous meta-analyses have indicated that the left prefrontal cortex (PFC) might be predominantly involved in verbal WM processes [40,108]. In previous fMRI studies, the PFC showed significant left-hemispheric lateralization during a verbal WM task and right lateralization during a spatial WM task [109].

Since the present study used auditory and visual verbal stimuli during the n-back task, and the evidence that in the processing of speech signals, binaurally presented stimuli elicit more robust brain responses in the left hemisphere [106,110], we expected that the brain would demonstrate significant left-hemispheric lateralization during task performance. This working expectation was confirmed. In fact, the almost significant difference in gamma activation between hemispheres suggests a left-hemispheric localization of VWM as proposed in the literature [40,41], regardless of deafness. Moreover, our results fit well with a previous PET study that showed that PFC activity in younger adults was left-lateralized for verbal WM stimuli [111]. So, the trend of greater gamma activation in the left hemisphere found for the VWM task independently of groups could indicate left-hemispheric lateralization of the cognitive function of auditory and visual VWM. Clearly, this is a result to be re-evaluated with a larger sample. However, this preliminary data could suggest that the cortical development of deaf children with preverbal cochlear implantation maintains the same lateralization pathway as the HC as far as the VWM. It could probably be the result of the support given by the preverbal CI that allows for maintaining brain plasticity, supporting the hypothesis of a sensitive period [112,113,114] for the stabilization of the integration of sensory stimuli. In fact, it is well known by EEG studies that children who received CI at an early age (<3.5 years of age at fit) showed activation of the auditory cortical areas contralateral to their cochlear implant, which resembled that of hearing subjects [115,116]. Moreover, in our data, the absence of differences in the activation of the auditory and visual area between HC and UCI argues for the lack of audio-visual cross modalities in CI users, suggesting that earlier and longer CI use would inhibit the cross-modal reorganization of auditory regions in early deafness as hypothesized by Ding and colleagues [39]. Furthermore, the comparison between HC and UCI concerning gamma activation in the left hemisphere would show that verbal WM is stronger characterized by gamma left hemisphere activation only in HC (in line with our previous work, see [28]). In fact, there were no significant differences in the other EEG frequencies in the left hemisphere between groups and within the UCI, which seem to be deficient in gamma activation during the n-back task, whereas less gamma pattern of activity is considered indicative of (inefficient) neural resource management to achieve proper cognitive performance [117]. Moreover, studies showed specific processing deviances in individuals with language and literacy problems in processing gamma rates [118,119].

The interpretation of results in terms of gamma inactivation in UCI would seem to be expressly validated during audio VWM by the activations found in the parietal area in the control group. In fact, in the parietal area, the HCs were significantly less active in the most challenging audio task than in the other conditions (Figure 5). Moreover, in addition to a strong relationship between parietal gamma activation and IES for audio conditions, we found that the first variable predicted the second (Figure 8), suggesting that gamma-band-sustained increase over parietal sites is involved with a role in WM maintenance and the binding of auditory memory representations during the n-back, in line with what was found during a different visual memory task, in the posterior—occipital areas [120,121]. These data could lead to the conclusion that gamma activation in the parietal area for auditory stimuli is the neurophysiological support for task performance only in HC children suggesting that CIs children could require greater overall recruitment of neural resources to respond similarly to the control group.

Overall, our findings confirm the role of gamma-band oscillations as candidates for the working memory function. Consistently, numerous studies observed the involvement of the gamma band in the perception and maintenance of the WM [120,121,122]. Moreover, functionally, gamma oscillatory activity is thought to participate in integrating neural networks within and across brain structures, facilitating coherent sensory registration [123].

Additionally, the integration of binaural input occurs via coincident counters in the superior olivary complex -SOC [124] and this brainstem region is involved in the identification of the angle and location of the sound source and the difference between the time and intensity of sounds reaching each the ear [125]. Moreover, as unilateral implant use causes abnormal reorganization of the auditory pathway at the level of the brainstem and the cortex [126], the possible key role played by SOC in achieving adaptive changes in auditory processing [127] and the evidence that SOC is the first nucleus in the central auditory pathway that receives auditory information from both ears [128], globally lead to the hypothesis that the absence in UCI of the pattern of significant parietal activation in gamma for the 2 audio condition highlighted in HC could be due precisely to the failure to achieve the processing of the binaural signal in the SOC. Their clinical condition of unilateral implanted (although all preverbal see Table 1) is in line with studies showing that monolateral deafness results in substantial changes in neural activity from the subcortical to the central auditory system [129,130] and could, in fact, result in a deficit of processing the acoustic signal and then in the non-activation of gamma-band as a result of the deficient passage of the auditory signal in the SOC. The relationship found in gamma activation with the current age and not with the auditory age nor with behavioral data (IES; ACC) could mean that the UCIs, although they have developed the brain processing that leads to the representation of the stimulus in the cortex in a similar way to the hearing person (who conversely did not show a correlation between gamma levels and age, therefore suggesting the occurred reaching of a “plateau”), would seem to assume their need for additional time to develop the same pattern of gamma activation for auditory VWM. However, this EEG pattern could be definitive due to unilateral cochlear implantation. Comparing subjects with two cochlear implants may reveal which of the two interpretations may be correct. Moreover, an assessment with auditory brainstem response (ABR) that provides information concerning the functional integrity of brainstem nuclei [131] may or may not offer support for the hypothesis of partial maturation of the SOC and thus its hypoactivation resulting in reduced gamma-band activation. However, such an analysis was not a stated purpose of the present study, but these assumptions may offer insights for further investigation.

The regression analysis conducted on the UCI group finally confirms that the chronological age predicted just parietal gamma activity for audio VWM and is not predictive for video conditions (Figure 9). Therefore, overall, the regression model seems to confirm the existence of an electroencephalographic pattern supporting VWM performance for auditory stimuli, a pattern that, however, needs to reach a certain level of brain maturation to be configured in a unilateral deaf child brain.

## 5. Conclusions

This is the first study investigating differences in neurophysiological patterns between hearing and UCI children during a VWM task performed in two modalities (visual and auditory). We obtained promising positive results that include the absence of differences in both behavioral performance and neurophysiological indicators of attention (frontal theta) and workload (WI) between the clinical group and controls. Although restricted to a small sample, these results can confirm the effectiveness of clinical and rehabilitation treatments for children with unilateral cochlear implants. However, the different recruitment between HC and UCI found in the parietal area for the auditory stimulation appears to be closely related to the representation of the senses at a cortical level and the different relationships found between EEG oscillations and the behavioral and biological data (current age) between HC and UCI, leading to the conclusion that the difference in activation in gamma frequency in the parietal areas can be the proper support to the auditory VWM. Thus, the synergy (absent in children with CI) between gamma activation and performance could be the reason for the extreme variability found in psychological assessments of VWM tasks in previous studies. Further studies exploring the heterogeneity of characteristics of CI users (e.g., age at implantation, comparing pre- and post-implanted groups; different etiology of onset of deafness) could provide additional support for our findings and open up future directions of investigation.

Finally, our findings, although preliminary and needing further investigations in larger samples, support evidence that EEG may hold promise in uncovering the neurophysiological mechanisms underlying the variability in VWM outcomes between HC and UCI. They may provide evidence of the activation of gamma in the parietal areas as a possible signature of neurophysiological support for auditory VWM, and to open both new lines of research on purely behavioral data, and extend existing and future rehabilitation pathways.

## Figures and Tables

**Table 1:** Demographic and clinical data concerning the unilateral cochlear implant (UCI) group. In particular: onset of deafness, its etiology, and auditory age (years of cochlear implant use since implantation).

Participants	Gender	Onset of Deafness	Degree	Aetiology	Current Age	Age at CI	Auditory Age
P1	F	Congenital	Profound	Homozygous mutation of the connexin-26 gene	12.00	2.90	9.09
P2	F	Congenital	Profound	Homozygous mutation of the connexin-26 gene	10.73	1.86	8.87
P3	F	Congenital	Profound	Homozygous mutation of the connexin-26 gene	11.49	1.41	10.07
P4	F	Congenital	Profound	Homozygous mutation of the connexin-26 gene	11.49	1.41	10.07
P5	F	Congenital	Profound	Homozygous mutation of the connexin-26 gene	11.14	1.16	9.97
P6	M	Congenital	Profound	Usher syndrome	11.58	0.79	10.78
P7	F	Congenital	Profound	Unknown	10.09	1.79	8.30

**Table 2:** n-back task behavioral performances of both investigated groups (unilateral cochlear implant users—UCI; hearing control—HC) and the total participants (TOT) in terms of accuracy (ACC) and inverse efficiency score (IES) expressed in milliseconds (ms) for all the experimental conditions.

ACC (%) and IES (ms) for n-Back Task Conditions								
		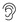	ACC Audio 0-back	ACC Audio 1-back	ACC Audio 2-back	IES Audio 0-back	IES Audio 1-back	IES Audio 2-back
**Groups**	UCI		97.62%	82.65%	75.17%	559.83	814.71	1140.49
	HC		97.67%	86.67%	86.20%	660.45	830.72	1018.99
	TOT		97.06%	85.01%	81.66%	619.02	824.13	1069.02
		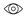	ACC Video 0-back	ACC Video 1-back	ACC Video 2-back	IES Video 0-back	IES Video 1-back	IES Video 2-back
**Groups**	UCI		94.90%	81.29%	80.61%	447.78	875.87	894.68
	HC		90.00%	81.67%	74.52%	505.95	738.62	1079.56
	TOT		92.02%	81.51%	77.03%	482.00	795.13	1003.43

## Data Availability

The data presented in this study are available on request from the corresponding author.

## Abbreviations

ABR	Auditory Brainstem Response
ACC	Accuracy
ANOVA	Analysis of Variance
AOI(s)	Area of Interest(s)
AUD	Audio
CIs	Cochlear Implants
CRs	Correct Responses
DHH	Deaf and Hard-of-Hearing
EEG	Electroencephalogram/Electroencephalography
EFs	Executive Functions
fMRI	Functional Magnetic Resonance Imaging
HC(s)	Hearing Control(s)
Hz	hertz
IAF	Individual Alpha Frequency
IES	Inverse Efficiency Score
ISI	Interstimulus Interval
m	media
ms	millisecond
PA	Parietal Area
PE	Proportion of Errors
PFC	Prefrontal Cortex
PET	Positron Emission Tomography
PSD	Power Spectral Density
RPM	Raven’s standard Progressive Matrices
RTs	Reaction Times
SD	Standard Deviation
SM	Sensory Modality
SOC	Superior Olivary Complex
UCIs	Unilateral Cochlear Implant Users
VIS	Visual
WISC V	Wechsler Intelligence Scale for Children Fifth Edition
VWM	Verbal Working Memory
WI	Workload Index
WL	Mental Workload
WM	Working Memory
